# Supplementary material for: Using the TSA-LSTM two-stage model to predict cancer incidence and mortality
Source: PLoS One. 2025 Feb 20;20(2):e0317148. doi: 10.1371/journal.pone.0317148 (PMC11841919; doi:10.1371/journal.pone.0317148)
Supplement: S1 Appendix — (DOCX) [file pone.0317148.s001.docx]

**Appendix 1: Centers for Disease Control and prevention (CDC)**

<https://www.cdc.gov/cancer/uscs/public-use/> and American Cancer Society

<https://www.cancer.org/research/cancer-facts-statistics/all-cancer-facts-figures/cancer-facts-figures-2020.html>
