# Supplementary material for: Using the TSA-LSTM two-stage model to predict cancer incidence and mortality
Source: PLoS One. 2025 Feb 20;20(2):e0317148. doi: 10.1371/journal.pone.0317148 (PMC11841919; doi:10.1371/journal.pone.0317148)
Supplement: S2 Appendix — (DOCX) [file pone.0317148.s002.docx]

**Appendix 2: Linear, Quadratic, or Cubic data**

| **Year** | **Linear Spline** | **Quadratic Spline** | **Cubic Spline** | **Actual Data** |
| --- | --- | --- | --- | --- |
| 1990 | 132829.15 | 132829.15 | 132829.15 | 132829.15 |
| 1991 | 135214.57 | 135913.55 | 136614.52 | 136715.81 |
| 1992 | 139032.46 | 139534.58 | 140432.53 | 140521.80 |
| 1993 | 143184.56 | 143478.57 | 144587.32 | 144690.28 |
| 1994 | 147871.67 | 147871.67 | 147871.67 | 147871.67 |
| 1995 | 148165.28 | 149735.63 | 150639.32 | 150939.31 |
| 1996 | 151099.65 | 151577.58 | 152399.65 | 152599.66 |
| 1997 | 153344.94 | 153344.94 | 153344.94 | 153344.94 |
| 1998 | 151715.67 | 152258.37 | 153658.37 | 153758.37 |
| 1999 | 153693.86 | 153545.08 | 154542.08 | 154642.08 |
| 2000 | 154607.93 | 155109.73 | 156109.83 | 156609.83 |
| 2001 | 159546.46 | 159546.46 | 159546.46 | 159546.46 |
| 2002 | 161902.58 | 162402.58 | 163002.58 | 163402.58 |
| 2003 | 165775.88 | 166475.88 | 166975.88 | 167275.88 |
| 2004 | 170639.91 | 170639.91 | 170639.91 | 170639.91 |
| 2005 | 172776.65 | 173476.65 | 174076.65 | 174276.65 |
| 2006 | 175573.30 | 176373.30 | 177173.30 | 177573.30 |
| 2007 | 182245.83 | 182245.83 | 182245.83 | 182245.83 |
| 2008 | 186361.55 | 187061.55 | 187561.55 | 187861.55 |
| 2009 | 191627.41 | 192727.41 | 193327.41 | 193527.41 |
| 2010 | 198137.30 | 198137.30 | 198137.30 | 198137.30 |
| 2011 | 200381.11 | 201381.11 | 202081.11 | 202381.11 |
| 2012 | 206419.34 | 206419.34 | 206419.34 | 206419.34 |
| 2013 | 208565.56 | 209065.56 | 210265.56 | 210565.56 |
| 2014 | 214658.17 | 214658.17 | 214658.17 | 214658.17 |
| 2015 | 236123.99 | 236123.99 | 236123.99 | 236123.99 |
| 2016 | 259736.38 | 259736.38 | 259736.38 | 259736.38 |
| 2017 | 285710.02 | 285710.02 | 285710.02 | 285710.02 |
| 2018 | 314281.03 | 314281.03 | 314281.03 | 314281.03 |
| 2019 | 345709.13 | 345709.13 | 345709.13 | 345709.13 |
| 2020 | 380280.04 | 380280.04 | 380280.04 | 380280.04 |
| 2021 | 418308.04 | 418308.04 | 418308.04 | 418308.04 |

Note: Outcomes expected divided by 100.
