# Supplementary figures and images for: Using the TSA-LSTM two-stage model to predict cancer incidence and mortality
Source: PLoS One. 2025 Feb 20;20(2):e0317148. doi: 10.1371/journal.pone.0317148 (PMC11841919; doi:10.1371/journal.pone.0317148)

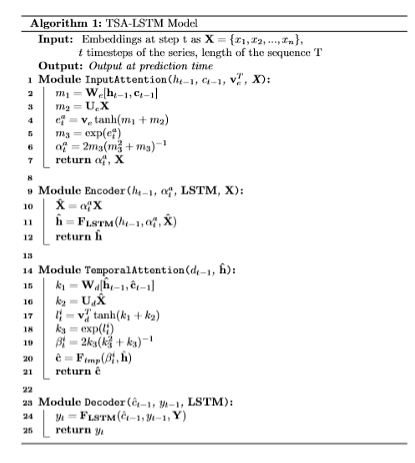

Supplement: S3 Appendix — (PNG) [file pone.0317148.s003.png]
