# Supplementary material for: Using the TSA-LSTM two-stage model to predict cancer incidence and mortality
Source: PLoS One. 2025 Feb 20;20(2):e0317148. doi: 10.1371/journal.pone.0317148 (PMC11841919; doi:10.1371/journal.pone.0317148)
Supplement: Attachment — Submitted filename: Response to Reviewers-TSLM-2024.docx [file pone.0317148.s005.docx]

**Authors' Response to Reviewers' Comments**

PONE-D-23-37813

Using the TSA-LSTM Two-Stage Model to Predict Cancer Incidence and Mortality

PLOS ONE

Dear editor and reviewers,

We express our sincere gratitude for your correspondence and the insightful feedback provided by the reviewers about our paper titled " **Using the TSA-LSTM Two-Stage Model to Predict Cancer Incidence and Mortality” (PONE-D-23-37813).** The feedback provided is invaluable and conducive to enhancing our work and furnishing the necessary direction for our research. We have meticulously reviewed and revised the paper in response to the recommendations provided by the editor and reviewers. The responses to the blue-highlighted questions in the comments are provided in the following point-by-point list.

**Response of editor and reviewers’ response**

Additional Editor Comments:

Having carefully gone through the manuscript, I find the subject matter both relevant and timely. Your approach to using the TSA-LSTM model for predicting cancer incidence and mortality is innovative and could potentially contribute significantly to the field. However, I have a few questions that I believe, once addressed, could further strengthen the paper:

Model Validation and Comparison: Could you provide more details on how the TSA-LSTM model's predictive performance compares to existing models? Specifically, it would be beneficial to see comparisons to other prevalent models in cancer incidence and mortality prediction, focusing on accuracy, reliability, and computational efficiency.

**Reply:** Your insightful remarks and recommendations are much appreciated. We have meticulously changed it in accordance with your insightful remarks. With insightful feedback, we have meticulously rewritten the paper point by point.

**Response:** The heading 1, second paragraph, and third paragraph, as well as heading 2.5, first paragraph, and 3.1, first paragraph, have been altered to reflect the updated and revised model comparison and validation.

Data Preprocessing and Feature Selection: Your paper mentions data preprocessing and sample expansion design. Could you elaborate more on the feature selection process for the TSA-LSTM model? How were the variables chosen, and what was the rationale behind including or excluding certain variables?

**Reply:** Your insightful remarks and recommendations are much appreciated. We have meticulously changed it in accordance with your insightful remarks. With insightful feedback, we have meticulously rewritten the paper point by point.

**Response:** The first and third paragraphs of heading 3.1 have been revised and updated to reflect the aforementioned queries and to illustrate the method for selecting variables.

Interpretability of Model Results: The TSA-LSTM model seems to be a complex structure. How do you address the challenge of interpretability of the model's results, especially for healthcare professionals who might not have a deep understanding of machine learning models?

**Reply:** Your insightful remarks and recommendations are much appreciated. We have meticulously changed it in accordance with your insightful remarks. With insightful feedback, we have meticulously rewritten the paper point by point.

**Response:** Revisions and updates have been made to the first paragraph of heading 4.2 and the third paragraph of heading 4.3.

Practical Applications and Limitations: Finally, could you discuss the practical applications of your model in real-world healthcare settings? Are there any specific limitations or challenges in implementing this model in a clinical environment?

**Reply:** Your insightful remarks and recommendations are much appreciated. We have meticulously changed it in accordance with your insightful remarks. With insightful feedback, we have meticulously rewritten the paper point by point.

**Response:** Practical application-related changes have been made to Heading 5, and the study's specific limitations have been described in the first and second paragraphs.

**1^st^ Reviewer's Responses to Questions**

Reviewer #1: The research is interesting; however, this paper is suggested to be published in PLOS ONE after authors have addressed the comments listed below:

1. For the literature review, authors should refer to more recent research. The current references are not up to date. Authors need to refer to more ISI/Scopus research work instead of the online/conference resources.

**Reply:** Your insightful remarks and recommendations are much appreciated. We have meticulously changed it in accordance with your insightful remarks. With insightful feedback, we have meticulously rewritten the paper point by point.

**Response:** In the literature review, sections 2.1 to 2.5 have been updated and amended to include the most recent sources in accordance with the objectives of the concern study.

2. The literature review need to be further improve. The current review is too general and lengthy. The literature review need to be more specific and need to be tie back to the domain of the research. Please provide a summary at the end of the section to summary the research insights.

**Response:** The entirety of section 2 (literature review) has been changed and updated to reflect the most recent research. Second, each subsection addresses the subject and aims in a more deliberate and comprehensive manner. Third, the summary at the end of each section has been revised and updated.

3. Authors need to identify the research gap and highlight the contribution(s) of the research work in the manuscript to shows the novelty of the research. The proposed methodology existed in the literature, please highlight the novelty of the research work in the manuscript.

**Response:** The gaps in research and the contributions that have been highlighted have been updated and placed in the fourth paragraph of heading one. These changes deeply concern the primary contribution of the study, which is the research gap and the novelty of the study.

4. Authors should compare the proposed techniques with the state-of-arts to further prove the contribution(s)/novelty of the research work.

**Response:** The research gaps and contributions that were identified as the foundation for the proposed study technique have been amended and appended to the fifth paragraph of the first heading. The modifications have a substantial effect on the primary contribution of the study, which is the discovery of a research gap and the uniqueness of the study.

5. More scientific reasoning should be added in the experimental results' explanations.

**Response:** In general, the experimental fourth section has been thoroughly reviewed and amended, including the headings 4.2 and 4.3 (first and second paragraphs), 4.8 (first and eighth paragraphs), and the first and second paragraphs of the fifth component of the research (discussion).

**2. Reviewer's Responses to Questions**

Reviewer #2: The literature review was thorough and covered all the background information needed to understand the work in this paper.

**Reply:** Your insightful remarks and recommendations are much appreciated. We have meticulously changed it in accordance with your insightful remarks. With insightful feedback, we have meticulously rewritten the paper point by point.

**Response:** The entire section on the literature has been rewritten and updated, and the most recent research findings have been incorporated for the purpose of providing additional clarification. A number of changes and updates have been made to sections 2.1 to 2.5 in order to incorporate the most recent sources in accordance with the goals of the concern research.

The results and the subsequent discussion were interesting, specially with the different deep learning algorithms and their performance on the dataset.

**Response:** Specifically, Sections 4 and 5 have been updated and reviewed with a greater degree of consciousness, and all of the experimental data have been carefully examined, including the most recent research investigations. Particularly noteworthy are headings 4.2, 4.3, and 4.8, in addition to the full section of discussion.

There were a few minor grammatical errors, but nothing major.

**Response:** The entire text, from section 1 to section 6, is checked twice and revised in accordance with the instructions that were provided. Additionally, with the assistance of Grammarly, the manuscript was finally checked and the many issues were resolved.

As per the given instruction, I have separated the policy recommendation portion with new ideas and concluded the study output with modified technology. Such as in this context, exploring the new alternative energy resources. The replacement of fossil fuel infrastructure will take the form of developing renewable energies, such as thermal, hydroelectric, and wind power plants, which are more than welcome. Clean energy also reduces the impact of gas extraction and coal mining. This study forwards the most relevant policy recommendations according to the above results.

The given reference I have included in my study.

**Thank you very much again for your valuable comments and suggestions.**
