# Supplementary material for: Using the TSA-LSTM two-stage model to predict cancer incidence and mortality
Source: PLoS One. 2025 Feb 20;20(2):e0317148. doi: 10.1371/journal.pone.0317148 (PMC11841919; doi:10.1371/journal.pone.0317148)
Supplement: Attachment — Submitted filename: Response to Reviewers-TSLM-2024-04-1 (A)-(Second).docx [file pone.0317148.s006.docx]

**Authors' Response to Reviewers' Comments**

PONE-D-23-37813

Using the TSA-LSTM Two-Stage Model to Predict Cancer Incidence and Mortality

PLOS ONE

Dear editor and reviewers,

We express our sincere gratitude for your correspondence and the insightful feedback provided by the reviewers about our paper titled " **Using the TSA-LSTM Two-Stage Model to Predict Cancer Incidence and Mortality” (PONE-D-23-37813).** The feedback provided is invaluable and conducive to enhancing our work and furnishing the necessary direction for our research. We have meticulously reviewed and revised the paper in response to the recommendations provided by the editor and reviewers. The responses to the blue-highlighted questions in the comments are provided in the following point-by-point list.

**Response of editor and reviewers’ response**

**Reviewer's Responses to Questions**

Reviewer #4: 1.The cancer data are known to be complex and heterogeneous. How well does the TSA-LSTM Two-Stage Model handle such complexities and heterogeneity?

**Reply:** Your insightful remarks and recommendations are much appreciated. We have meticulously changed it in accordance with your insightful remarks. With insightful feedback, we have meticulously rewritten the paper point by point.

While the TSA-LSTM Two-Stage Model exhibits promise in the analysis of cancer data, its limitations must be taken into account, particularly when confronted with the complex landscape of cancer research. This manuscript delves into additional details, including these. We demonstrate the positive aspects of this study, including the benefits of two-stage learning and sequential data (Heading 2.6, third paragraph). Additionally, substantial obstacles must be addressed in regards to interpretability (heading 4.5, fourth paragraph) and overfitting (headings 4.4 and 4.5, first paragraph). Finally, when employing advanced techniques, we utilize Long Short-Term Memory (LSTM) and Recurrent Neural Network (RNN) models, which have exceptional capabilities for processing sequential data and revealing intricate relationships that are hidden within time series. Long-sufficiency-term (LSTM) models, which fall under the category of RNNs, demonstrate remarkable effectiveness in handling data dependencies that persist for a prolonged period of time.

2.Does the methodology have any limitations or have it been made considering of some assumptions that could possibly lessen the accuracy and/or generalizability of the outputs?

**Reply:** Your insightful remarks and recommendations are much appreciated. We have meticulously changed it in accordance with your insightful remarks. With insightful feedback, we have meticulously rewritten the paper point by point.

The potential efficacy of the TSA-LSTM Two-Stage Model in the analysis of intricate data is contingent upon the information employed being of sufficient quality and comprehensiveness. Moreover, the accuracy of the model may be constrained by assumptions regarding the fundamental data structure and its ability to apply to new, unobserved data. Implementing the model to its utmost potential for tasks such as cancer analysis requires meticulous data management, investigation of alternative models, and continuous research. Furthermore, the first and fourth paragraphs of heading 3.1 have been revised, and the limitations of the study have been briefly explained in the first and second paragraphs of heading 5.

3. Have you ever been to a live concert of your favorite band playing your favorite songs and experienced the sound coming out of state-of-the-art sound systems that lasts your entire drive home?

**Reply:** Your insightful remarks and recommendations are much appreciated. We have meticulously changed it in accordance with your insightful remarks. With insightful feedback, we have meticulously rewritten the paper point by point.

Sorry sir I have no idea of this question, might be its mistakenly appeared.

4.Where did the cancer data come from and how were the process and the quality controlling assurance implemented to ensure the data was correct and reliable?

**Reply:** Your insightful remarks and recommendations are much appreciated. We have meticulously changed it in accordance with your insightful remarks. With insightful feedback, we have meticulously rewritten the paper point by point.

The dataset came from the Centers for Disease Control and prevention (CDC) and the American Cancer Society (Appendix 1). The data includes cancer incidence rates, risk factors such as tobacco smoking, alcohol consumption, physical inactivity, obesity/excess body weight, and cancers caused by these risk factors. Our 1990-2021 data spans 32 years (Heading 3.1). The American Cancer Society and the CDC's USCS program both utilize a comprehensive strategy to ensure the integrity of their cancer data. To begin with, data is collected from a variety of sources, including hospitals and state-run cancer registries, thereby reducing the potential influence of errors that are unique to any one system. Coding and standardized reporting formats guarantee uniformity throughout this enormous data pool. Inconsistencies and information gaps that have been identified are thoroughly addressed via a combination of automated and manual quality checks. Statistical analysis incorporates the consideration of potential biases, and the data is accompanied by comprehensive metadata that ensures transparency regarding its limitations and collection methodologies. Although these rigorous quality control procedures are delayed, both organizations are consistently working to improve their data collection and analysis techniques, thereby establishing their cancer statistics as a reliable resource for the general public and researchers.

5.How did the statistical analysis specifically indicate the missing, unreliable or unpleasant data?

**Reply:** Your insightful remarks and recommendations are much appreciated. We have meticulously changed it in accordance with your insightful remarks. With insightful feedback, we have meticulously rewritten the paper point by point.

In case of statistical analysis, the TSA-LSTM model is effective for assessing sequential cancer data but is very dependent on the quality of the data. Statistical analysis serves as a diligent companion, detecting potential problems (Heading 3.1, 3,4, and 5 paragraph). In this study we pinpoint outliers or missing information that may be impeding the model's learning by examining its prediction mistakes and difficulties with certain data points (Heading 4.1 and 4.2,4.4-4.7). Moreover, outlier detection and analysis of loss functions can reveal inaccurate data points in the cancer dataset. This statistical investigation ensures that the TSA-LSTM model is trained using the most precise information available. When evaluating the model's outputs, the emphasis is on comprehending trends rather than sensationalizing "unpleasant" discoveries. These trends can help direct more research or public health actions focused on enhancing cancer outcomes.

6.Was there some difficulty united the heterogeneous datasets with varying origins?

**Reply:** Your insightful remarks and recommendations are much appreciated. We have meticulously changed it in accordance with your insightful remarks. With insightful feedback, we have meticulously rewritten the paper point by point.

When employing the TSA-LSTM model or any other machine learning model for cancer research, this is indeed a significant challenge: unifying heterogeneous datasets of varying provenance. Data preprocessing (heading 3.1) and data transformation techniques were utilized in this study to surmount these obstacles. Much like the preprocessing of data. To guarantee that the combined dataset is consistent and appropriate for the TSA-LSTM model, this is a critical phase requiring techniques such as feature engineering (generating new features from existing ones), normalization (managing missing values and inconsistencies), and data cleaning (addressing missing values and inconsistencies). Additionally, within the realm of data transformation techniques, normalization and standardization can be implemented to standardize the magnitudes of all features. This standardization enables the TSA-LSTM model to efficiently acquire knowledge from the merged data.

7.In what way the predictive precision of the two-stage model of TSA-LSTM is better than other models which are called the state-of-the-art of cancer prediction.

**Reply:** Your insightful remarks and recommendations are much appreciated. We have meticulously changed it in accordance with your insightful remarks. With insightful feedback, we have meticulously rewritten the paper point by point.

When asserting that the TSA-LSTM Two-Stage Model unequivocally surpasses all other cutting-edge models for cancer prediction, extreme caution must be exercised. Current research is examining the comparative efficacy of the TSA-LSTM Two-Stage Model and other approaches, as it is a relatively novel method. Although it exhibits potential, further research and rigorous comparisons are required prior to admonishing its superiority.The particular predictive task determines the efficacy of every model, including the TSA-LSTM. While it may demonstrate exceptional proficiency in forecasting tumor growth patterns, its performance may be comparatively subpar when confronted with more complex inquiries such as treatment response prediction. This Model exhibits notable capabilities in processing sequential data and has the potential to discern intricate relationships enclosed within the data. On the contrary, alternative cutting-edge models may possess distinct advantages, such as enhanced precision in identifying particular breeds of cancer or image categorization for tumor identification. Finally, by capitalizing on its capability to analyze sequential data and potentially perceive intricate relationships within the data, the Model exhibits promise as a valuable instrument for cancer prediction. However, this is a subject of continuous investigation, and depending on the specific form of cancer and the influencing factors, other cutting-edge models may be more appropriate for particular prediction tasks (Heading 3.1, 3.3).

8.Was there suitable evaluation metrics used with the models' output, e. g. sensitivity, specificity, ROC or precision -recall curves?

**Reply:** Your insightful remarks and recommendations are much appreciated. We have meticulously changed it in accordance with your insightful remarks. With insightful feedback, we have meticulously rewritten the paper point by point.

The TSA-LSTM Two-Stage Model shows potential for cancer prediction by analyzing sequential data, but assessing its actual effectiveness necessitates a comprehensive evaluation. Researchers use a set of criteria such as sensitivity (accurately identifying cancer cases) and specificity (accurately categorizing cancer-free persons). This study use Common Data Interpolation Methods (heading 2.3), specifically Linear spline interpolation, to create straight lines between data points in table gaps, typically found in astronomical data. Precision-recall curves are especially useful in imbalanced datasets, such as those found in cancer research, where positive instances (cancer) are less frequent than negative cases. The measures, combined with comparisons to known benchmarks and the performance of other state-of-the-art models, offer a detailed insight into the strengths and drawbacks of the TSA-LSTM model. This thorough assessment method enables researchers to select the most appropriate model for a particular prediction job, pinpoint areas needing enhancement, and eventually enhance cancer prediction accuracy, resulting in improved patient outcomes.

9.Was there any data related difference in treatment of cancer through different cancer types or stratum?

**Reply:** Your insightful remarks and recommendations are much appreciated. We have meticulously changed it in accordance with your insightful remarks. With insightful feedback, we have meticulously rewritten the paper point by point.

According to heading 2, our historical cancer incidence data includes demographics, environmental variables, cancer diagnosis rate time series, and other crucial information that sets it apart from previous studies. Standardize, address missing values, and provide data windows suitable for LSTM. The TSA-LSTM model utilizes multiple LSTM cell layers to capture long-term dependencies in sequential data. Attention mechanisms in LSTM forecasting aid in concentrating on pertinent features. Create a two-stage method to pinpoint and enhance essential characteristics for accurate prediction. Assess the model's predictive performance by comparing expected values with actual observations. Revise the model to enhance performance and address problems. When assessing model estimates, take into account epidemiology, historical trends, and variables related to cancer risk. TSA-LSTM employs attention mechanisms and LSTM networks in a two-stage framework to accurately predict cancer occurrence and understand its intricate temporal trends. By utilizing advanced deep learning techniques, researchers and data scientists can better cancer risk assessment, optimize resource allocation, and inform public health policy.

10.Does the TSA-LSTM Two-Stage Model model depict the crucial factors and can provide information about the trends in cancer incidence and mortality rates?

**Reply:** Your insightful remarks and recommendations are much appreciated. We have meticulously changed it in accordance with your insightful remarks. With insightful feedback, we have meticulously rewritten the paper point by point.

The capability of the TSA-LSTM Two-Stage Model to identify patterns in sequential cancer data is exceptional. Consequently, it becomes an invaluable instrument for discerning temporal variations in cancer incidence and mortality rates among diverse populations. The model, however, fails to shed light on the underlying reasons for these trends. Operating as an advanced detective, it identifies patterns without necessarily identifying the perpetrators. In order to derive a more comprehensive comprehension of the pivotal factors that propel these trends, scholars supplement the model's outputs with conventional statistical analysis methods. Moreover, it is critical to integrate the knowledge and proficiency of cancer epidemiologists and biologists. Their expertise in the disease and its fundamental biology enables them to analyze the patterns and propose possible etiological elements. By integrating the trend-spotting functionalities of the TSA-LSTM model with statistical analysis and domain knowledge, this integrated methodology provides a robust perspective on the field of cancer epidemiology. In conclusion, it has the potential to provide guidance for the advancement of prevention and treatment strategies that are more efficacious, thereby securing a more promising future for individuals afflicted with cancer.

11.How without difficulty understandable are the predictions generated by the version for healthcare practitioners and policymakers?

**Reply:** Your insightful remarks and recommendations are much appreciated. We have meticulously changed it in accordance with your insightful remarks. With insightful feedback, we have meticulously rewritten the paper point by point.

The interpretability of the TSA-LSTM model's predictions for healthcare practitioners and policymakers walks a tightrope between simplicity and technical accuracy. Ideally, this tailored version would translate the model's complex predictions into easily digestible insights. Imagine user-friendly dashboards displaying the probability of a tumor growing by a specific percentage within a defined timeframe. However, achieving this level of crystal-clear interpretability in deep learning models can be challenging. A more realistic scenario might involve the model offering predictions alongside explanations that, while not revealing every intricate detail, provide a sufficient understanding of the rationale behind the results (heading 4.4). Ultimately, the key lies in close collaboration between data scientists, statisticians, and healthcare professionals. By presenting the information in a clear, concise, and actionable way, even if not entirely transparent, this user-friendly version of the TSA-LSTM model can empower healthcare practitioners and policymakers to navigate the complexities of cancer data. This, in turn, can lead to more informed decision-making, paving the way for improved cancer prevention and treatment strategies (heading 6, second paragraph).

12. Were efforts made to interpret the learned representations or function significance inside the model?

**Reply:** Your insightful remarks and recommendations are much appreciated. We have meticulously changed it in accordance with your insightful remarks. With insightful feedback, we have meticulously rewritten the paper point by point.

There is a significant probability that endeavors were undertaken to interpret the learned representations or ascertain the function significance within the TSA-LSTM Two-Stage Model. This is particularly noteworthy in light of the fact that the healthcare practitioner and policymaker versions of the model require a certain degree of comprehension regarding the factors that impact their predictions. In a medical setting, it would be futile to use a model that is wholly opaque to inform decisions. In general, despite the uncertain nature of the TSA-LSTM model's interior workings, it is probable that there are endeavors underway to enhance interpretability, specifically with regard to the healthcare-oriented iteration. It is critical to regard the model's predictions as a practical instrument rather than a conclusive resolution. When it comes to ensuring optimal patient care, clinical expertise and domain knowledge will perpetually be of the utmost importance.

Thirteen.
13.What capacity clinical programs should get up from the predictions generated through the TSA-LSTM Two-Stage Model?

**Reply:** Your insightful remarks and recommendations are much appreciated. We have meticulously changed it in accordance with your insightful remarks. With insightful feedback, we have meticulously rewritten the paper point by point.

The capacity that clinical programs should derive from the TSA-LSTM Model's predictions is context-dependent and prediction-specific. In general, the model possesses the capacity to augment clinical programs through the provision of valuable insights pertaining to cancer progression, risk, and potentially personalized treatment planning. Nevertheless, it is imperative to bear in mind the constraints of the model and supplement its prognostications with clinical knowledge and other pertinent data to ensure the highest quality of patient care.The following are some particular capabilities that clinical programs may derive from the model:

- Risk stratification involves determining which patients would benefit from more intensive treatment or closer surveillance in accordance with their predicted risk of progression.
- Assist in the identification of patients who possess particular tumor characteristics and could potentially benefit from targeted therapies or clinical trials.
- Resource allocation decisions can be informed by forecasting potential increases in the incidence of cancer in particular populations or regions.

Potentially, clinical programs can optimize resource allocation and patient outcomes by judiciously and ethically applying the model's outputs.

14.Has the version been validated in medical settings, and in that case, what have been the consequences?

**Reply:** Your insightful remarks and recommendations are much appreciated. We have meticulously changed it in accordance with your insightful remarks. With insightful feedback, we have meticulously rewritten the paper point by point.

The potential of the TSA-LSTM Two-Stage Model to improve clinical programs is contingent upon its ability to be successfully validated. Due to its recent emergence, this approach has not yet achieved widespread adoption in clinical settings. It is critical to conduct thorough testing in order to verify that the model exhibits satisfactory performance when applied to actual patient data and can generalize beyond the scope of the training data (heading 3). A validation that is effective has the potential to revolutionize cancer care. Envision healthcare professionals leveraging the insights provided by the model to optimize resource allocation, risk stratification, and personalization. It may also facilitate the patient selection process for clinical trials or targeted therapies. Negative validation results may, nevertheless, have disadvantages (heading 4). Misapplication of forecasts or erosion of confidence in AI-powered healthcare may ensue. Utilization that is transparent and ethical is vital. Despite the model undergoing validation, its limitations must be explicitly stated, and clinical expertise must consistently take precedence. The transformative capacity of the TSA-LSTM Two-Stage Model in clinical practice is contingent upon its comprehensive validation and conscientious integration into the healthcare domain.

15. What are the results of the model's predictions for healthcare resource allocation, prevention strategies, or remedy planning?

**Reply:** Your insightful remarks and recommendations are much appreciated. We have meticulously changed it in accordance with your insightful remarks. With insightful feedback, we have meticulously rewritten the paper point by point.

According to heading 4, the TSA-LSTM Two-Stage Model could improve healthcare resource allocation, prevention, and cure planning, but it needs confirmation. This new model isn't yet embedded in clinical settings. Imagine a world where the model's insights allocate resources. Predicting cancer hotspots could help healthcare organizations allocate doctors, nurses, and equipment. Preventive measures may help. The model may identify high-risk individuals who should receive screens or lifestyle modifications. Most importantly, remedy planning seems promising. Individual tumor analysis by the model could lead to individualized treatment plans. Clinicians could use the model's insights to choose the most effective medications or radiation dosages or identify patients for targeted therapy or clinical trials. Caution is advised. These are theoretical applications, thus validation is key. The model is good at spotting trends, not certainties, so clinicians must interpret them for each patient. The healthcare practitioner version must also explain its forecasts to develop trust and integrate into clinical decision-making. Finally, ethical questions about resource equity and model output biases cannot be ignored. The TSA-LSTM Two-Stage Model could transform healthcare, but it needs extensive validation and prudent adoption. Whether this approach delivers will be determined in the coming years.

16.Were there any moral considerations addressed in the development or deployment of the TSA-LSTM Two-Stage Model, along with privacy issues or bias mitigation?

**Reply:** Your insightful remarks and recommendations are much appreciated. We have meticulously changed it in accordance with your insightful remarks. With insightful feedback, we have meticulously rewritten the paper point by point.

According heading 3 and 4 the development of the TSA-LSTM Two-Stage Model involves more than mere technical expertise. Its development and implementation incorporate ethical considerations. It is essential to mitigate bias in order to prevent the model from exacerbating healthcare disparities. Probably to resolve this, techniques implement fairness metrics and guarantee diverse training data. Strict regulations are adhered to with regard to the anonymization and security measures implemented to safeguard patient data privacy. In addition, a healthcare practitioner version that is unambiguous and simple to comprehend is crucial for establishing confidence and guaranteeing the ethical incorporation of the model's prognostications into clinical decision-making. Ensuring fair and equal distribution of the resources it facilitates and the advantages it provides is of the utmost importance. The potential for the TSA-LSTM Two-Stage Model to transform into a potent instrument for a healthcare system that is both equitable and efficient can be realized by taking into account these ethical considerations.

17.How would possibly the usage of predictive fashions in most cancers prediction effect healthcare disparities or get admission to to care?

**Reply:** Your insightful remarks and recommendations are much appreciated. We have meticulously changed it in accordance with your insightful remarks. With insightful feedback, we have meticulously rewritten the paper point by point.

According to heading 4, regarding healthcare disparities, predictive models for cancer prediction, such as the TSA-LSTM Two-Stage Model, navigate a fine line. Although biased training data can further widen the disparities in misdiagnosis and resource allocation, these gaps can be mitigated with the help of the models. Consider the potential of early detection in underserved regions, where the model could identify high-risk individuals who might fail screenings otherwise. Personalized treatment plans that are customized to the unique attributes of each individual have the potential to enhance outcomes for all. By concentrating prevention efforts on high-risk populations, the cancer burden in disadvantaged communities could be reduced. During the development process, diverse datasets and impartiality metrics are essential for bridging the equity gap. Furthermore, it is crucial to guarantee fair and equal access to the technology and the healthcare resources that it facilitates the allocation of. By prioritizing equity and ensuring that all individuals have an opportunity to benefit, these models can be transformed from potential disparity wideners to potent instruments for a healthcare system that is more accessible and equitable.

18. Are there any accidental outcomes or dangers related to counting on algorithmic predictions for healthcare choice-making?

**Reply:** Your insightful remarks and recommendations are much appreciated. We have meticulously changed it in accordance with your insightful remarks. With insightful feedback, we have meticulously rewritten the paper point by point.

According heading 2.5, 2.8 (1^st^ paragraph) algorithmic forecasts in healthcare, like those produced by the TSA-LSTM model, provide optimism for the future of tailored medicine but should be used carefully. While they can offer valuable insights into the probability, progress, or effectiveness of cancer therapy, their internal workings may remain obscure. Clinicians may struggle to trust the model's logic and incorporate its results appropriately. According heading 3.1, 3.3 some patient groups can face unfair outcomes because of hidden biases in the training data. Excessively pretending these outcomes may devalue the crucial skills and experience of a physician. Moreover, there is still a possibility of inaccurate positive or negative outcomes, leading to unnecessary medical treatments or missed diagnosis. According to heading 4.5 and 4.6, enhance patient care, it is crucial to view algorithmic forecasts as a supplementary tool rather than a definitive solution. Understanding the model's limitations clearly and being transparent are crucial. To make the best healthcare decisions, it is crucial to combine predictions with clinical judgment and the medical history of each patient.

In accordance with the directive, I have partitioned the policy recommendation segment into novel concepts and concluded the study's results with modified technologies. For instance, investigating the newest alternative energy sources in this context. The infrastructure formerly reliant on fossil fuels will be replaced by the construction of wind, hydroelectric, and thermal power facilities, all of which are greatly appreciated. Additionally, the utilization of clean energy mitigates the consequences of gas extraction and coal mining. The aforementioned findings inform the policy recommendations that are most pertinent to this study. I have incorporated the reference provided into my research.

Again, many thanks for your insightful remarks and recommendations.
