# Supplementary material for: Using the TSA-LSTM two-stage model to predict cancer incidence and mortality
Source: PLoS One. 2025 Feb 20;20(2):e0317148. doi: 10.1371/journal.pone.0317148 (PMC11841919; doi:10.1371/journal.pone.0317148)
Supplement: Attachment — Submitted filename: Response to Reviewers-TSLM-2024-06-01 (Third & Fifth).docx [file pone.0317148.s007.docx]

**Authors' Response to Reviewers' Comments**

PONE-D-23-37813

Using the TSA-LSTM Two-Stage Model to Predict Cancer Incidence and Mortality

PLOS ONE

Dear editor and reviewers,

We express our sincere gratitude for your correspondence and the insightful feedback provided by the reviewers about our paper titled " **Using the TSA-LSTM Two-Stage Model to Predict Cancer Incidence and Mortality” (PONE-D-23-37813).** The feedback provided is invaluable and conducive to enhancing our work and furnishing the necessary direction for our research. We have meticulously reviewed and revised the paper in response to the recommendations provided by the editor and reviewers. The responses to the blue-highlighted questions in the comments are provided in the following point-by-point list.

**Response of editor and reviewers’ response**

**Reviewer's Responses to Questions**

**Reviewer #3:**

**PONE-D-23-37813R2**

**Using the TSA-LSTM Two-Stage Model to Predict Cancer Incidence and Mortality**

**PLOS ONE**

**Reviewer #3:** This paper proposed a TSA-LSTM two-stage model to predict cancer incidence and mortality. The idea is interesting and the research is complete. Some improvements should be made before the paper is accepted.
1. The length of the paper should be limited. The author needs to condense the paper and emphasize the key content.

**Reply:** Your insightful remarks and recommendations are much appreciated. We have meticulously changed it in accordance with your insightful remarks. With insightful feedback, we have meticulously rewritten the paper point by point.

**Answer:** The entire manuscript was rewritten and checked on a regular basis. In every single paragraph, read it over once more and try to reduce as much as you can.

2. The content of Appendix 4 and 5 should be exchanged. Besides, both of them are crucial components of the paper, which should be shown in the main body of the paper.

**Reply:** I appreciate your intelligent comments and advice. In response to your informative comments, we thoroughly altered it. Our informed input helped us painstakingly rewrite the article.

**Answer:** Appendices 4 and 5 have been updated and amended, and the language has been modified heading 4.2 and 4.4 to reflect these changes.

3. The results are not rigorous enough since the average score is not convincing. As a prediction method, RMSE and R2 should be listed to evaluate the performance. Besides, boxplots of errors are also suggested to be provided.

**Reply:** Your insightful remarks and recommendations are much appreciated. We have meticulously changed it in accordance with your insightful remarks. With insightful feedback, we have meticulously rewritten the paper point by point.

**Answer:** Changes and modifications have been made to the final paragraph of heading 4.1.

**PONE-D-23-37813R2**

**Using the TSA-LSTM Two-Stage Model to Predict Cancer Incidence and Mortality**

**PLOS ONE**

**Reviewer #5:**

Reviewer #5: I am sorry to say that I have no idea what the authors intended or did in this long text. For example, the introduction mentions predicting disease outcomes, as "However, surgeons struggle most with illness outcome prediction," the data used are not data from which illness outcomes can be predicted, and no results or discussion of illness outcome prediction are given. The section titled as "review," it should be based on a systematic literature review process, or at least the literature search method should be described in the method section.
I believe that the authors aimed to develop a model used deep learning for projections of cancer incidence. I believe that the authors should focus on the aim and results.

**Answer:** Thank you so much for your perceptive comments and suggestions. Your wise observations have been carefully considered, and we have made the necessary changes. We have rewritten the document from the ground up in response to the helpful comments. My work has been edited and updated in accordance with the suggestions made above.

p.3-6 Introduction
What the authors are doing is prediction of the future incidence of cancer. I suggest that the authors focus on that and describe which aspects of the methods currently used to cancer incidence prediction need to be improved, and the advantages of the authors' new methods.

**Reply:** Your insightful remarks and recommendations are much appreciated. We have meticulously changed it in accordance with your insightful remarks. With insightful feedback, we have meticulously rewritten the paper point by point.

**Answer:** With the addition of new cited research, the **third paragraph** of **heading 1** has been modified in accordance with the valuable recommendation.

p.7-13 2. Literature Review
What is presented in this section is hardly to title as review, so it would be appropriate to delete repetition and shorten it to be an introduction. If you want to make it as a review, you should either conduct a systematic review or describe how you selected the papers to be reviewed in the method section.

**Reply:** Your insightful remarks and recommendations are much appreciated. We have meticulously changed it in accordance with your insightful remarks. With insightful feedback, we have meticulously rewritten the paper point by point.

**Answer:**

2.2 Description of Tableau
I do not understand the intent of describing Tableau. The authors do not use Tableau in their cancer incidence and mortality prediction model (Results of prediction for mortality were not shown in this draft.though.). Tableau seems to be used only to find factors to be included in the model or to visualize the results, there is no those figure or table though. If so, it is necessary to describe how the use of Tableau improves the search of risk factor and visualization of the result. For the usage in current draft, a sentence "Tableau was used to create the graph" in the method section is sufficient.

**Reply:** Your insightful remarks and recommendations are much appreciated. We have meticulously changed it in accordance with your insightful remarks. With insightful feedback, we have meticulously rewritten the paper point by point.

**Answer:** The informative comments and suggestions that you have provided are very much appreciated. Your informative comments together with the observation that Tableau enhances the search for risk factors and the depiction of the results have been taken into careful consideration and incorporated into the changes that we have made (Section 2.2, last paragrah). As a result of receiving intelligent input, we have painstakingly reworked the document paragraph by paragraph.

p.13-17, 3. Empirical Methodology
The dataset came from the Centers for Disease Control and prevention (CDC) and the American Cancer Society (Appendix 1).
Not only the description of the data were obtained from the Centers for Disease Control and prevention (CDC) and the American Cancer Society but also more information, for example, is it based on a registry system, what year to year incidence rates, when was the information on risk factors collected, for what populations, and by what methods?, etc., should be described. Brief description and citation of the article or website where they are described is possible. It is very important information what data were used for the model building and validation. This information help the readers to understand the results the authors obtained.

**Answer:** I would want to express my gratitude for the insightful remarks and recommendations that you have supplied. We have diligently rewritten the paper paragraph by paragraph as a consequence of obtaining insightful feedback, and Section 3, heading 3.1-3 have been edited and updated as a result of this.

Figure 2.
There seems to be a lack of understanding of what the diagram created in Tableau shows; in Text, this diagram is used to show how bar charts can be easily created by drag-and-drop in Tableau, but for that purpose, it would be appropriate to show the Tableau startup screen and the operating procedure, I do not believe that it is the authors' aim to explain how to use Tableau, so I think that this diagram is NOT necessary.

**Answer:** As a result of receiving insightful input, we have meticulously rewritten the paper paragraph by paragraph, and Section 4, heading 4.1-7 have been edited and updated as a consequence of this. The paper has also been updated and modified.

Figure 3.
The y-axis is the incidence rate, and the notation of the x-axis and the legend are the same. This figure could be lead misunderstanding of readers that Tableau does not support such an easy adjustment. There is also a discrepancy between the figure and the text.

**Answer:** 4.3 headings in the third and fourth paragraphs have been updated and altered to include the most recent research ideas.

Figure 4.
Figure 4 seems shows the attributable risk of obesity to the incidence rate for each cancer. It is natural that breast cancer, which has a high incidence rate, would be larger, and the graph does not show what the authors intended. The wording also needs to be considered, since being related alone does not mean being the cause. It is a basic requirement of scientific papers that the appropriate units must be indicated on the graphs, so please do not forget to do so.

**Answer:** 4.3 headers have been revised and updated to include the most recent research concepts. The last two paragraphs have also been affected.

Figure 5.
The color shading and the height of the bars represent the same thing. It is well known that ninety percent of cervical cancers are caused by HPV (https://www.cdc.gov/hpv/parents/cancer.html), and 100% of cervical cancers are never caused by obesity.

**Answer:** In order to incorporate the most recent research principles, the headings for section 4.3 have been altered and updated. Furthermore, the final paragraphs have been impacted as well.

**Reply:** The informative comments and suggestions that you have provided are very much appreciated. Your perceptive comments have been taken into consideration, and we have made the necessary adjustments. As a result of receiving intelligent input, we have painstakingly reworked the document paragraph by paragraph.

Again, many thanks for your insightful remarks and recommendations.
