# Supplementary material for: Using the TSA-LSTM two-stage model to predict cancer incidence and mortality
Source: PLoS One. 2025 Feb 20;20(2):e0317148. doi: 10.1371/journal.pone.0317148 (PMC11841919; doi:10.1371/journal.pone.0317148)
Supplement: Attachment — Submitted filename: Response to Reviewers-TSLM-2024-10-26 .docx [file pone.0317148.s008.docx]

**Authors' Response to Reviewers' Comments**

PONE-D-23-37813

Using the TSA-LSTM Two-Stage Model to Predict Cancer Incidence and Mortality

PLOS ONE

Dear editor and reviewers,

We express our sincere gratitude for your correspondence and the insightful feedback provided by the reviewers about our paper titled " **Using the TSA-LSTM Two-Stage Model to Predict Cancer Incidence and Mortality” (PONE-D-23-37813).** The feedback provided is invaluable and conducive to enhancing our work and furnishing the necessary direction for our research. We have meticulously reviewed and revised the paper in response to the recommendations provided by the editor and reviewers. The responses to the blue-highlighted questions in the comments are provided in the following point-by-point list.

**Response of editor and reviewers**

**Additional Editor Comments:**

Your paper has some issues with the figure's quality. The figures are blurry and not readable. Figure 2 Visual on creating a visualization in Tableau, the label seems not complete.  And the proposed TSA-LSTM design is blurry and not readable at all.

**Reply:** We are grateful for your insightful comments and recommendations. We have made the necessary adjustments to the paper in accordance with your feedback. The paper has been meticulously rewritten point by point.

The entire manuscript has been thoroughly reviewed, with a particular emphasis on figure 2. The figure was previously blurry and unreadable due to its size, but I have since replaced it with the actual figure. I have attempted to modify the figure with the assistance of PACE, but it remains blurry. Consequently, the original figure has been incorporated into the manuscript. Additionally, the final paragraph of heading 4.2 has been updated. I am grateful for the comments and suggestions of all the reviewers, as they have been extremely helpful in the process of modification. Thank you again for your insightful remarks and recommendations.
